# Symmetry-breaking phase transitions in highly concentrated semen

**DOI:** 10.1098/rsif.2016.0575

**Published:** 2016-10

**Authors:** Adama Creppy, Franck Plouraboué, Olivier Praud, Xavier Druart, Sébastien Cazin, Hui Yu, Pierre Degond

**Affiliations:** 1 Université de Toulouse, INPT, UPS, IMFT (Institut de Mécanique des Fluides de Toulouse), Allés Camille Soula, 31400 Toulouse, France; 2 CNRS, IMFT, 31400 Toulouse, France; 3 INRA, CNRS, UMR, 37380 Nouzilly, France; 4 CNRS, Institut de Mathématiques de Toulouse UMR 5219, 31062 Toulouse, France; 5 Department of Mathematics, Imperial College London, London SW7 2AZ, UK

**Keywords:** active fluids, self-organized hydrodynamics, Vicsek model, semen quality

## Abstract

New experimental evidence of self-motion of a confined active suspension is presented. Depositing fresh semen sample in an annular shaped microfluidic chip leads to a spontaneous vortex state of the fluid at sufficiently large sperm concentration. The rotation occurs unpredictably clockwise or counterclockwise and is robust and stable. Furthermore, for highly active and concentrated semen, richer dynamics can occur such as self-sustained or damped rotation oscillations. Experimental results obtained with systematic dilution provide a clear evidence of a phase transition towards collective motion associated with local alignment of spermatozoa akin to the Vicsek model. A macroscopic theory based on previously derived self-organized hydrodynamics models is adapted to this context and provides predictions consistent with the observed stationary motion.

## 1. Introduction

Biological fluids in physiological contexts are usually confined. For most of them, confinement is a hindrance to flow owing to viscous dissipation. However, occasionally, when a supplementary external active mechanism is involved, confinement can help (e.g. in microciliary beating, peristaltic waves, etc.). Active fluids, i.e. fluids composed of a suspension of swimming microorganisms, is a new area of research where unexpected behaviours have been discovered (see reviews [1,2] and [3]). For example, in the case of dilute suspensions of pushers (resp. pullers), i.e. microswimmers that push (resp. pull) the fluid ahead, such as spermatozoa (resp. chlamydomonas), a decreasing [4] (resp. increasing [5]) apparent viscosity for increasing suspension concentration is found. In the case of pushers [6] and more specifically spermatozoa [7–10], recent observations have suggested that interactions with the boundaries might be key to guide spermatozoa towards the oviduct tract, or generate vortices in confined bacterial suspensions [11]. Similar unexpected effects have recently been observed in systems of colloidal rollers [12,13] confined in microfluidic arenas.

**Figure 1.**
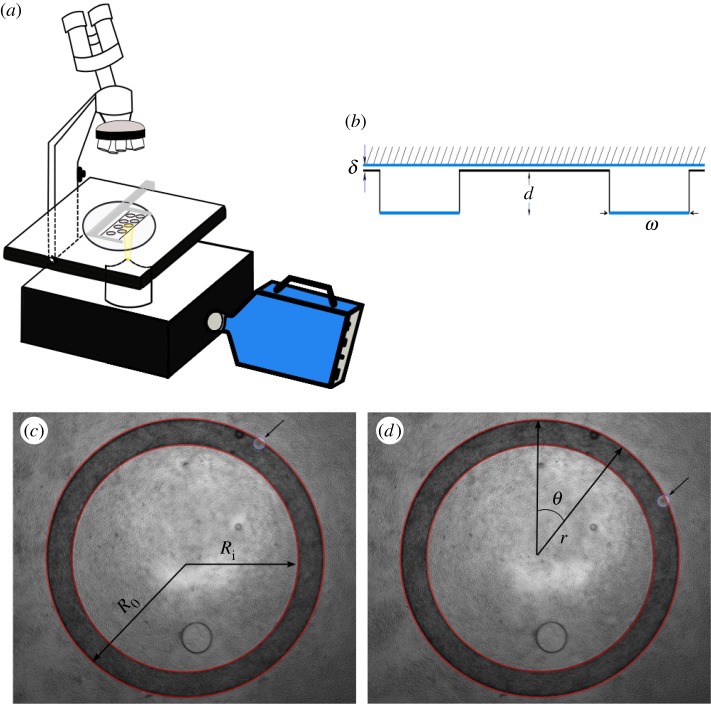
(*a*) Sketch of the experimental set-up composed of a 16 bits high speed camera sCMOS 2560 × 2160, with a pixel size of 6.5 µm^2^ on captor mounted on a phase contrast microscope. (*b*) Sketch of the lateral view of the annulus geometry. Blue colour defines the glass substrate, whereas black represents the SU-8 resin film. (*c,d*) Display a top snapshot of the real annulus obtained with a ×4 optic within a time lapse of 3 s. One can observe (cf. arrows) the displacement of a dust particle convected along the flow by the semen rotation. (Online version in colour.)

**Figure 2.**
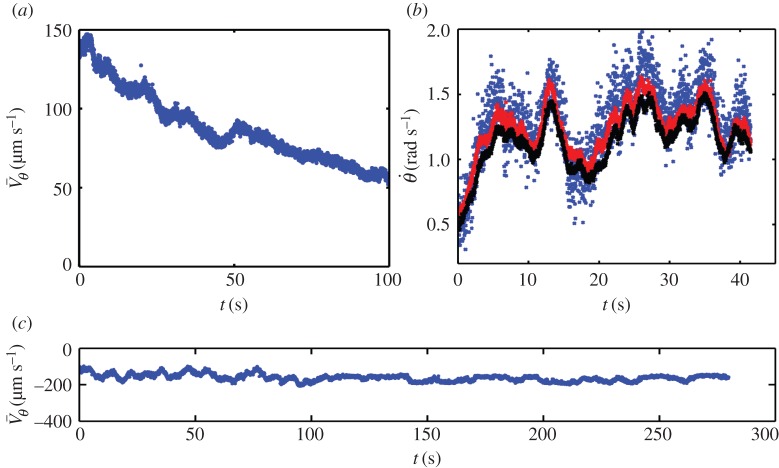
Time variation of the average spinning velocity 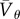. All three panels display the collective motion measurement in the annulus for pure sperm. (*a,c*) Exemplify two distinct long-time slow decay of the average spinning velocity and (*b*) validates the measurement method at a given radius location. More precisely in (*a*) a slow, almost linear decay on long times is observed. The spermatozoa concentration is 2.25 billion ml^−1^. (*b*) Comparison of semen azimuthal angular velocity 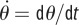 at a given radial position in the ring measured with three different methods: PTV (blue dots), one-dimensional PIV (red dots) and two-dimensional PIV (black dots). (*c*) Time variation of the spinning velocity 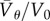 for a sample sustaining rotation over long times. The sample consists of pure semen having spermatozoa concentration of 3.7 billion ml^−1^. (Online version in colour.)

According to Wioland *et al*. [11], spontaneous vortex motion observed in *B. subtilis* suspensions can be explained by the interplay of boundary curvature, steric and hydrodynamic interactions. Similar large-scale coherent dynamics in sessile drops of bacterial flows [14] or semen [15,16] were indeed reported many years ago. In the case of semen, the analysis of collective motility (also known as mass motility (MM)) based upon the observed turbidity in the vicinity of the sessile drop contact line has been used as a reliable index for male fertility scoring [16] in livestock. The MM score has been used for 30 years in animal insemination centres to estimate semen quality [17]. It is a score on a scale of 1–5 (the value 1 corresponds to an almost steady turbidity and the value 5 when it displays a large number of rapid and active whirlpools). Other emergent states in active swarms have been reported [18–20] associated with macroscopic ordered states owing to local orientation effects. Here, we show that a fresh semen sample confined inside a ring (figure 1*b* shows its lateral view) displays a very robust and stable rotational motion (cf. figure 2*c*) which is uniform along the azimuthal direction. We subsequently study it.

## 2. Material and methods

### 2.1. Experiments

#### 2.1.1. Microfluidic chip fabrication

The annulus has been patterned with a photolithography technique inside a clean room facility, using a polymer film layered onto a glass substrate. A SU-8 polymer film has first been coated onto a glass wafer [21,22], using a solvent dissolved injection technique. Using an adapted photo mask designed to prevent adhesion and cracking problems caused by residual stresses, a standard UV lithography has then been performed. The quality of the resulting patterning was *a posteriori* quantitatively controlled with a microscope. Various designs for the ring have been tested by varying the polymer thickness, i.e. the depth *δ* of the annulus from 25, 60 to 100 µm, the average diameter of the ring (0.5, 1, 2 and 3 mm) as well as its width *w* (50–200 µm).

#### 2.1.2. Microscopy measurements

We use a BH-2 Olympus microscope with a 4× phase contrast objective. The microscope field depth has been estimated to be 25 µm, and the focal plane is centred in the middle between the top and bottom walls of the annulus, as in [23]. Most of the reported experiments are considering annulus depth equal to *δ* = 100 µm, so that the reported measurements are safely away from the top and bottom walls.

Note that the current experimental set-up does not permit to visualize the interactions between cells and the possible mechanisms by which clusters of cells form. This indeed would provide invaluable insights into the alignment mechanisms (which is at the basis of the model) but is unfortunately unrealizable with the current techniques. First, the imaging performed here is a phase contrast microscopy that does not allow individual motion to be detected. Furthermore, the microscope focal depth is about 30 µm, so that the observed signal integrates the contribution of several layers of cells. Hence, individual motion cannot be identified. Finally, from some previous staining experimental tests that we have conducted, we have observed that fluorescent staining of such concentrated suspensions (volume fraction is almost 50%) does not allow us to distinguish individual cells from the bright background generated by many close sources of light. Hence, only a moderate fraction of stained cells could possibly be imaged individually. In this case, it would not be possible to quantify alignment with neighbours (as many neighbours would be unstained and thus not observable). One could possibly imagine alternative microscopy techniques, e.g. sheet planar illumination microscopy, but one would then need to re-assess the entire experimental set-up, which is beyond the scope of this study.

#### 2.1.3. One-dimensional particle image velocity method

In this section, we explain how we have designed a dedicated method to perform a fast and reliable computation of the vortex state (VS) velocity, associated with the azimuthal rotation of the fluid inside the annulus. Starting from a high frame-rate imaging of the annulus, providing grey-level pixels *I_t_*(*x*, *y*) at time *t* on a Cartesian grid, the following first steps have been coded.

(i) Detect the circular edges of the annulus.
(ii) Find the centre of the circle, the inner and the outer radius of the annulus.
(iii) Define a regular grid in polar coordinates grid 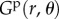 with discrete set of points along *r* and *θ*.
(iv) Interpolate *I*(*x*, *y*) into 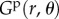.

We have transformed the annulus radial geometry into a longitudinal one, making it a virtual straight channel. We are only interested in the displacement of the fluid along *θ*. The idea is then to use the polar representation to compute a one-dimensional version of the particle image velocity (PIV) method in order to extract the azimuthal velocity components 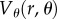 from the flow. For this, we wish to detect the correlation peak of two successive images 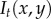 and 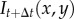 rotated along *θ*. For this, we first evaluate 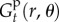 and 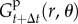. Then, instead of a direct computation of the correlation, using a convolution product of 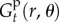 with 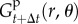 along the *θ*-direction, we evaluate this correlation with a fast Fourier transform (FFT) in the conjugate *k_θ_* Fourier space. This is done by the following steps.

(v) Compute the FFT 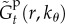 and 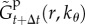, for each discrete *r* on the polar grid.
(vi) Compute the direct product
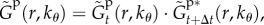
where asterisk denotes the complex conjugate.
(vii) Find the wavelength 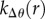 for which 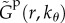 is maximum 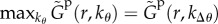. A one-dimensional Gaussian regression [24] is used to find the maximum 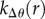 in order to provide subpixel accuracy.
(viii) Compute 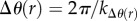.
(ix) Then, compute the angular velocity along *θ*,
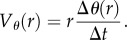
;

A standard average is performed over the discrete cylindrical grid to compute the average velocity from the inner radius *R*_i_ to the outer one *R*_o_2.1
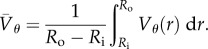
;

#### 2.1.4. Two-dimensional particle image velocity reconstruction

A standard image correlation PIV algorithm (Davis™) has been used on grey-level phase contrast images. The projection of the Eulerian velocity field in the focal plane is reconstructed on a two-dimensional grid, using a cross correlation PIV algorithm. The algorithm estimates the most probable displacement of small windows (identification window), centred at each node of the grid, between two successive frames. Iterative multigrid subpixel calculations are done that take into account the deformation of the identification window in order to increase accuracy and reduce peak locking. The spatial resolution is then dictated by the size of this window which, in our case, is 16 pixels × 16 pixels and which corresponds to 17 × 17 µm, because the pixel size is 1.08 µm. This size is small enough to resolve spatial structures of the order of a few spermatozoa. The PIV computation is restricted to the annular gap (*R*_i_ < *r* < *R*_o_), and the resulting velocity field of 4600 vectors is obtained on a Cartesian grid.

#### 2.1.5. Particle tracking velocimetry

The semen was seeded with poly(methyl methacrylate) beads of 1–20 µm diameter and density of 1.19 g cm^−3^ with a round and uniform shape (Dantec Dynamics, FPP-RhB-10). The characteristic time, *τ*_t_, needed for tracers to attain velocity equilibrium with the fluid using Stokes drag law is given by 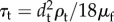, where *d*_t_ is the diameter and *ρ*_t_ is the density of the tracer particles and *μ*_f_ is the dynamic viscosity of the fluid. For 20 µm diameter tracers in water, *τ*_t_ ∼ 2.5 × 10^−5^ s, which is much shorter than the characteristic timescale of the flow. The settling velocity given by 
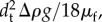
 where *g* is gravitational acceleration and Δ*ρ* is the density difference between the fluid and the tracers, is of order 2×10^−5^ m s^−1^ in water, much lower than the typical measured velocity of the fluid. In more viscous fluids such as seminal plasma, this settling velocity is even 50 times smaller, according to viscosity measurements [23]. Because these small beads are perfect inertia-less Lagrangian tracers of the flow, they offer the most reliable estimate of the local flow velocity, sampled at the particle scale. One should however expect some sampling velocity variation depending on the relative size between the bead and the active cells. If the bead size is comparable to the cells, then they cannot fit between the interstices of the cells, and might tend to move with the cells. If, on the contrary, they are much smaller than the cells, they should move with the fluid, and might be able to explore and sample the lubricated film dynamics between cells. Within the explored range of bead diameters, both scenarios exist. We indeed found variations between the resulting particle tracking velocimetry (PTV) velocity compared with PIV of almost 50% when considering beads smaller than cells; but dropping down to 25%, when considering the largest ones (cf. fig. 3 of Creppy *et al.* [23]). Considering that this difference was small enough to safely consider PIV measurements as a sensible estimate of the macroscopic velocity of the active suspension motion, we did not try to explore further fine-scale velocity features of the suspension. Finer attributes of the flow might nevertheless be obtained from a deeper exploration of the bead size dependence of PTV measurements. Analysing their spatial concentration patterns could also potentially probe cell concentration gradients. This might be an interesting track for possible experimental developments of this study.

**Figure 3.**
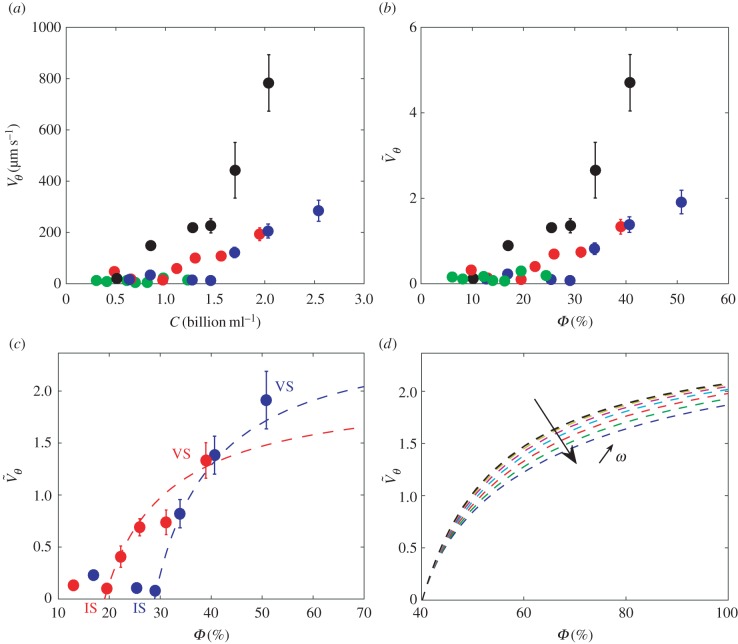
(*a*) Averaged spinning velocity 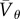 associated with the collective rotation of the semen versus sperm-cell concentration measured with spectrophotometry. (*b*) Dimensionless velocity 
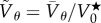
 (where 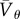 is the value provided in (*a*), and 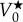 the average individual velocity obtained with a CASA suspended in seminal plasma) versus progressive spermatozoa volume fraction *Φ* (*Φ* is related to the measured concentration *C* provided in (*a*) from the known individual cell volume 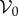, using 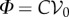). For both (*a*) and (*b*), each point represents a distinct experiment, and each colour is associated with the same semen, diluted into plasma. For each experiment, the velocity is averaged over 2000 frames for a 40 s recording. (*c*) Experimental results (circle symbols) fitted by the SOH model applied on an annular domain (dashed curves); (VS) stands for vortex state and (IS) for isotropic state, for which the average macroscopic velocity is zero. (*d*) Model prediction for the velocity as a function of the annulus width *w* 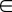 (50 µm, 700 µm). (Online version in colour.)

All particle detection algorithms have been coded in house, using Matlab software. Individual beads are tracked along time, using PTV of the fluorescent images that only show the tracer particles. Spatial resolution of the images is 1.5 µm.

*2.1.5.1. Detection.* Individual particles are first detected using a particle mask convolution analysis [25]. A particle template which consists of a two-dimensional Gaussian distribution *G* having an isotropic standard deviation of *σ* = 5 pixels is scanned over the entire image in order to detect the peaks of image intensity which then correspond to the particles, so that the normalized convolution reads2.2
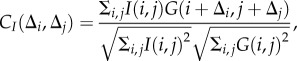
;where 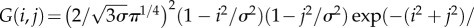 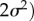 is the normalized wavelet, and *I*(*i*,*j*) is the original image grey-level intensity. A 0.45 threshold is then applied to 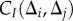 in order to provide isolated binary islands for each event detection. The positions of the centres of the detected particle images are then computed from performing a two-dimensional Gaussian regression [24] having an isotropic standard deviation of two pixels into the convolution field 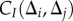. This procedure increases the contrast of the spherical particles, those being of known shape, so as to provide a subpixel accuracy to the evaluation of the position 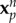 of particle *p* in image *n*.

*2.1.5.2. Tracking.* Because the mean displacement of the particle images is small compared with the mean interparticle distance, the matching particle pairs between two successive frames can be found by pairing each particle image in the first frame with its closest neighbour in the second frame. We consider that each detected position 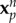 of image *n* has to be paired with an event of image *n* + 1, 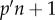 whose position 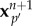 lies within a radius of *R* = 10 pixels from 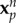, i.e. 
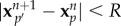
. The quantity *R* corresponds to the maximum displacement of a particle between two frames and is chosen after image observation. We then find the correct matching *p*′ for particle *p* for which the distance 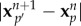 is minimum. Tracking a particle *p* whose position 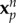 in image *n* varies to position 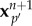 in the subsequent image *n* + 1 recorded Δ*t* later in the sequence permits one to determine its velocity 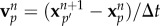.

#### 2.1.6. Comparison between particle image velocity in one dimension, particle image velocity in two dimensions and particle tracking velocimetry

We now report on comparisons between PIV and PTV measurements for one-dimensional velocity field azimuthal component. We refer to Creppy *et al.* [23] for comparisons in two-dimensional flows, as well as sensitivity with particle size. We investigate whether phase contract grey-level texture PIV velocity represents a sensible measurement of the real flow field from a direct comparison with PTV. We processed the recorded images with homemade Matlab one-dimensoinal PIV and PTV codes and with the two-dimensional correlation PIV algorithm (Davis™). As shown in figure 2*b*, one obtains a good relative agreement between PTV, one-dimensional PIV and two-dimensional PIV.

#### 2.1.7. Semen collection and mass motility score

Ejaculates from adult rams were collected by artificial vagina. Ejaculates were only accepted for this study when having an MM score of 3 or more and a thick and creamy appearance, free of blood and urine contamination. Wave motion of semen was observed in a 10 µl raw semen drop on the warming plate of a BH-2 Olympus microscope with a 4× phase contrast objective, and the MM score was performed based on Herman definition [17]. The MM score was refined from its original definition and scores subjectively assessed the rotation speed of dark waves (5 being the maximal speed). The sperm concentration *C* was also assessed by spectrophotometry. For the need of dilution, seminal plasma was prepared. For this, ejaculates were centrifuged at 10 000*g* for 10 min. The seminal plasma was collected and spun again (10 000*g*, 10 min) to remove any remaining sperm and cell debris. The seminal plasma was then pooled and frozen at 80°C until needed.

### 2.2. Model

Owing to the large sperm concentration *C* (about 5 × 10^9^ cm^−3^), associated with large volume fraction (generally above 20% and in some cases reaching 50%, close to the packing density), it is difficult to use mean-field approaches traditionally employed for lower density suspensions [26]. Indeed, classical expressions for hydrodynamic interactions between simple objects are based on far-field approximations (e.g. [3]). They cannot be used here, because the sperm cells are too closely packed and have complex moving shapes. So, we abandon the description of the fluid as the mediator of sperm–sperm interactions and consider a simple model which, we believe, correctly captures the phenomenology. Indeed, spermatozoa have elongated shape, and to be specific for ram, spermatozoa head size is 5 µm, flagellum length about 50 µm. Elongated self-propelled particles have been shown to align with their neighbours as a result of steric interactions [27–29]. However, sperm cells are pushers (i.e. propulsion results from fluid being pushed away behind the object). For such objects, the pure nematic alignment resulting from steric interaction (see corresponding models in [30–32]) cannot be used and must be replaced by polar alignment for which the relevant model is the Vicsek model [33] (see also [34] for a review on more general dry active matter models). Indeed, a configuration where two sperm cells are opposite head-to-head is unstable owing to the flagellum pushing, which results in alignment of the two sperm cells in both direction and orientation. Here, we use a continuum model derived from the Vicsek one, the self-organized hydrodynamic (SOH) model [35–38]. The rationale for using this model instead of the more classical Toner and Tu (TT) model [39] is developed below.

The Vicsek model [33] considers self-propelled particles moving at constant speed *V*_0_ and subject to (i) alignment with their neighbours within a sphere of radius *R* and (ii) directional noise. It exhibits phase transitions from disordered to aligned states when either the noise intensity is decreased or the density is increased. The Toner & Tu model [39] provides a macroscopic version of the Vicsek model derived on the basis of phenomenological symmetry considerations. By contrast, the SOH macroscopic model is a rigorous coarse-graining of the Vicsek model [35]. Its derivation starts from a mean-field equation (proved equivalent to the Vicsek model in the large particle number limit [40]) for the probability distribution 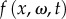 of the particle positions *x* and orientations *ω* at time *t*. At large spatio-temporal scales, the alignment intensity and noise are large, whereas the interaction radius *R* is small. Under this scaling, a previous study [35] shows that 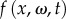 is given locally by a von Mises Fisher distribution 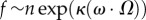, where *n* and *Ω* are the local particle density and mean orientation of the particles and ∼ recalls that there is an inessential normalizing factor. The concentration parameter *κ* is equal to the ratio of the alignment intensity and of the noise. The dependences of *n* and *Ω* upon (*x*, *t*) are shown to be solutions of the SOH model [35]:2.3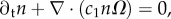;2.4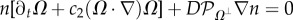;2.5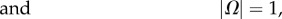;where *c*_1_, *c*_2_ and *D* are functions of the density *n*. The quantity 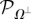 is the projection matrix normal to *Ω*, i.e. 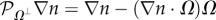. Equation (2.3) is the mass conservation equation and *V* = *c*_1_*Ω* is the mean sperm-cell velocity. Equation (2.4) describes the transport of the normalized mean velocity *Ω*(*x*, *t*). This transport results from (i) convection by the flow velocity (the term 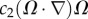) except that the convection speed is different from the flow speed by the factor *c*_2_/*c*_1_ ≠ 1 in general and (ii) influence of the pressure gradient (the term 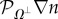). Thanks to the presence of 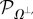 the normalization constraint on *Ω* (equation (2.5)) is satisfied at all times, provided that it is satisfied initially. Because the seminal fluid is neglected, the particle system does not obey momentum conservation (owing to self-propulsion) and lacks Galilean invariance, which is the reason for *c*_2_/*c*_1_ ≠ 1. Similar features are observed in other macroscopic models of self-propelled particles [41].

In [36], the dependences of the macroscopic parameters *c*_1_ and *c*_2_ upon *n* are related to how the microscopic alignment and noise intensities depend on the local particle alignment. In particular, for adequate choices of the model parameters, phase transitions as the density crosses a threshold *n*_c_ from disordered states (corresponding to *c*_1_(*n*) = *c*_2_(*n*) = 0 for *n* < *n*_c_) to aligned states (corresponding to *c*_1_(*n*), *c*_2_(*n*) > 0 for *n* > *n*_c_) can be obtained [36]. However, assumptions on the microscopic parameters translate into properties of the macroscopic parameters in a non-obvious way. Furthermore, it is experimentally almost impossible to measure the microscopic interaction parameters. So, we rather postulate the following formula for *c*_1_ and *c*_2_:2.6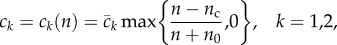;where 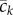 (as well as coefficient *D* in (2.4)) are estimated from previous work [37]. There are two free parameters *n*_0_ and *n*_c_ (the same for *k* = 1 and *k* = 2) which are used to fit the results with the experimental curves. Owing to the high variability of semen activity, each semen sample must be fitted independently. The convection speeds 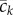 correspond to the limit of large densities where collective effects are well established. The critical density *n*_c_ corresponds to the onset of collective motion (indeed, when *n* < *n*_c_, *c_k_*(*n*) = 0 which corresponds to a state of zero average velocity, while when *n* > *n*_c_, *c_k_*(*n*) > 0, which corresponds to a state of finite average velocity), whereas *n*_0_ controls how strongly collective motion sets up near the critical density. The occurrence of second-order phase transitions with critical exponent 1/2 in the Vicsek model has been extensively discussed in the literature (e.g. [34]). Here, the parametrization (2.6), using a rational function, is preferred because it has finite slope controlled by the parameter *n*_0_ near the critical density *n*_c_. This feature allows a better match with the experimental data. Parameter *n*_0_ may be related with (so-far uncontrolled) individual-specific biological parameters such as semen rheology or semen protein content.

The SOH model bears analogies with the isothermal compressible Euler equations of gas dynamics, written as follows:2.7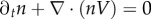;and2.8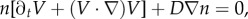;where *n* = *n*(*x*, *t*) and *V* = *V*(*x*, *t*) are the fluid density and mean velocity, respectively, and where we have assumed that the particle mass is equal to 1 for simplicity. Equations (2.7) and (2.8) express mass and momentum conservation, respectively. Equation (2.3) of SOH model also expresses mass conservation. Indeed, defining the flow velocity in the SOH model as *V* = *c*_1_*Ω*, equation (2.3) is identical with (2.7). Similarly, a term-by-term comparison of (2.4) and (2.8) shows a clear analogy between the two equations, the differences being brought by the necessity to preserve the normalization constraint (2.5) (hence the presence of the projection matrix 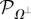) and by the different convection speeds *c*_1_ and *c*_2_ for *ρ* and *Ω*, respectively. The relation between the Euler and SOH model can be further expanded by noting that the latter is the limit of the former when a large relaxation force describing self-propulsion is added at the right-hand side. This modified system is written as2.9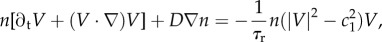;together with (2.7), where *τ*_r_ is a relaxation time and *c*_1_(*n*) > 0 is the self-propulsion speed averaged over a fluid element. The right-hand side tends to keep the speed |*V*| close to *c*_1_. The formal limit *τ_r_* → 0 of this model is proven in [42] to be the SOH model with *c*_2_ = *c*_1_ and the same coefficient *D* as in (2.9).

Most macroscopic models of dry active matter derive from the seminal work of Toner & Tu [39] and forthcoming works [43,44]. In its simplest form, the TT model is written [39]2.10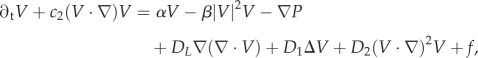;together with (2.7), with *α* < 0 and *c*_2_, *β*, *D_L_*, *D*_1_ and *D*_2_ all positive and *f* is a Gaussian random noise. Apart from the absence of the diffusion terms and of the random noise (those in the last line of (2.10)), the relaxed Euler model (2.7) and (2.9) is similar to the TT model (set *P*(*n*) = *D* log *n*, 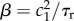, 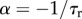, *c*_2_ = 1). Additional diffusion terms such as those of the TT model can also be obtained in the SOH model [45,46]. Here, because we are aiming at a simple qualitative agreement, they are ignored. Phase transitions in the TT model can be obtained upon postulating convenient dependence of the parameters on the density, similar to equations (2.6) for the SOH model. Among extensions of the TT model are those taking the fluid into account. These are known as ‘soft matter models’ for which a review can be found in [47]. For instance, in [11], the coupling of the Stokes equations for the fluid with a TT model for the active particles is performed. In [48], a derivation of the TT model is proposed. The approach differs from [35,36] in that the Vicsek model is replaced by a Boltzmann model describing binary interactions. However, the use of binary collisions for highly concentrated suspensions is subject to caution. Mathematically, this model is fairly challenging. For instance, the mere existence of non-isotropic stable equilibria is only proven when the noise distribution has only a finite number of non-zero Fourier coefficients [49], or when the noise is a Dirac delta [50]. A similar approach as in [48] is used in [12] to derive a macroscopic model for a population of motile colloidal beads moving under the action of an electric force. The rigorous derivation of such macroscopic models is still ahead of us. By contrast, the derivation of the SOH model from the mean-field Vicsek model is now equipped with a fully rigorous mathematical proof [51].

To reproduce the experimental geometry, we consider the SOH model (2.3)–(2.5) in a two-dimensional annular domain. We use polar coordinates 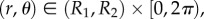 where *r* = |*x*|, *θ* is the polar angle and *R*_1_, *R*_2_ are the inner and outer radii of the annulus. Let 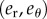 be the local basis associated with polar coordinates. Then, we let 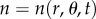 and 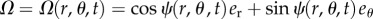, 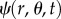 being the angle between *e*_r_ and *Ω*. In these cylindrical coordinates, the SOH model (2.1)–(2.3) is written2.11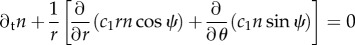;and
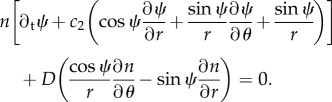
;The boundary conditions are such that *Ω* is tangent along the inner and outer circles (but different directions are possible at different points of the boundary), i.e. 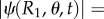 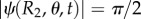.

Simple steady states of (2.11) and (2.12) take the form of a collective rotation. They are such that *n* is independent of *θ* and *ψ* is constant throughout the domain in one direction, for instance 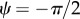 (clockwise rotation). Then, *n* = *n*(*r*) satisfies the simple ordinary differential equation2.13
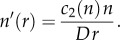
;Because the density is a key parameter in the experimental study, we fix the average density, namely
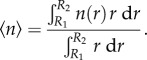
;To compare with the experimental findings, we compute the average azimuthal velocity, namely
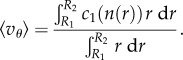
;It is readily seen that there are two cases.

(i) At low density, i.e. at low values of 〈*n*〉, we have *n* ≤ *n*_c_ for all 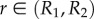. Then, *c_k_*(*n*) = 0, for *k* = 1,2. It results that 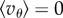, i.e. there is no net collective rotation of the sperm cells.
(ii) At larger densities, i.e. at larger values of 〈*n*〉, we can integrate the differential equation (2.13) and get2.14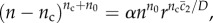;
where the constant *α* is determined from the prescribed value of 〈*n*〉. Solving (2.14) for *n* as a function of *r* with Newton's method, we can compute 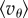.

The threshold value for 〈*n*〉 separating these two types of behaviour is precisely 〈*n*〉 = *n*_c_. The parameters 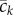 and *D* have physical dimension of a velocity and are measured in units of the individual sperm-cell velocity *V*_0_. The latter is provided by the experiments: *V*_0_ = 196 µm s^−1^ for the data coloured in red and *V*_0_ = 237 µm s^−1^ for those in blue in figure 3*c*. In the numerical simulations, we have taken the values *D* = 0.1 *V*_0_ and 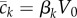 with *β*_1_ = 0.9486, *β*_2_ = 0.8486, estimated from previous work [37]. We identify the average density 〈*n*〉 to the experimental value of the volume fraction *Φ* (in per cent) and 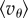 to the observed 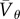, i.e.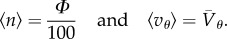;From the experimental data shown in figure 3*c*, we deduce an approximation interval for the values of (*n*_c_, *n*_0_) and then fit the data to obtain the best parameters, using a least-square method which we now describe. First, given (*n*_c_, *n*_0_), we compute the average density by solving equation (2.14) for the steady state *n*_s_ with an arbitrary value of *α*. Then, we find *α* corresponding to the volume fraction *Φ* in each data sample, compute 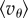 and compare it with the experimental value 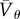. The fitting parameters (*n*_c_, *n*_0_) minimize the sum of the squares of the errors at the data samples which are shown by coloured discs in figure 3*c*.

We note that gradients of cell concentration are needed to produce the centripetal force that bends the particle orbits into circular ones. This is not a feature specific to the SOH model and such gradients would also be needed with other models such as the TT model. An estimate from (2.14) shows that the cell concentration at the outer cylinder is less than 50% larger than that at the inner cylinder. This variation of concentration is moderate and consistent with the existence of a small fraction of free space between the sperm cells. However, the model as presented here does not include repulsion by steric effects. When such effects are taken into account, like in [37], the last term of (2.4) involves the gradient of a pressure that depends nonlinearly on *n*. In this case, much smaller variations of *n* will result in large enough pressure gradients to provide the centripetal force needed to set the rotation motion. Here, these nonlinear effects have been ignored to keep the model as simple as possible. Sperm-cell velocity could be dependent on the density and velocities of the surrounding cells. Indeed, it is possible to envision that sperm cells swim relative to each other and that a high density of surrounding swimming cells would lead to an increase in sperm cell velocity. A first analysis of the dependence of swimming speed upon local density can be found in [52]. We could also envision that alignment is impeded at high density due to the lack of available free space for the particles to change their orientation. Density-dependent alignment frequencies have been previously considered in [53]: the corresponding macroscopic model is the SOH model with coefficients reflecting this additional dependence.

## 3. Results

### 3.1. Analysis of vortex state

Here, we show that a fresh semen sample confined inside a ring (figure 1*b* shows its lateral view) displays a very robust and stable rotational motion (cf. figure 2*a*,*c*) which is uniform along the azimuthal direction. The observed rotation that we subsequently refer to as a VS can unpredictably appear clockwise or counterclockwise.

When measuring the azimuthal velocity component 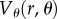 (cf. figure 1*d* for notations) of the velocity field of the semen during its spontaneous rotation using PIV techniques (cf. figure 2), we found very few spatial variations along the *θ* angle, consistently with a VS. By averaging it in the azimuthal direction along the ring as well as in its radial direction, we obtain a robust and precise estimate of the average rotation speed 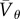 (cf. §2.1.3). We then perform a systematic measurement of the average rotation speed when varying the sperm cell concentration by diluting pure fresh semen into seminal plasma, at various dilution ratios, varying the concentration in a range of almost one decade.

This is illustrated in figure 3*a* for various semen samples. The volume fraction is deduced from concentration measurement from the known volume 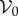 of spermatozoa using 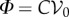. The volume 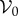 is estimated from an ellipsoidal head shape whose dimensions along the principal axes are 2, 4 and 6 µm. The flagellum, which is a thin, flexible, 50 µm long and 1 µm diameter cylinder is not included in this volume estimation because it is believed that steric interactions are mainly owing to head–head contacts. This estimation of the volume fraction makes comparisons with suspensions of dense spheroidal objects easier.

Both the concentration *C* and the volume fraction *Φ* are reported in figure 3. The concentration *C* is the quantity directly provided by the measurements, whereas the volume fraction *Φ* gives a sense of how close to the packing limit high-concentrated semen suspension are. For each semen sample, a phase transition between an isotropic state with no rotation at low concentration is followed by a spontaneous VS. The rotation speed increases with concentration after a threshold volume fraction *Φ*_c_ has been reached.

For each sample, a reference value for the individual velocity of the spermatozoon is measured, using a standard computer-aided sperm analysis (CASA) system by considering the average curvilinear velocity *V*_0_ over more than a hundred individual cells diluted into isotonic buffer. However, the rheology of isotonic buffer is close to that of water and differs significantly from that of seminal plasma. So, we also compare this measure with the average (CASA) curvilinear velocity of spermatozoon sampled from the same fresh semen while diluted in seminal plasma. The results are provided in table 1. One can observe that the ratio of the average curvilinear velocities in isotonic buffer and in seminal plasma lies in the range [0.61–0.88], with a 0.74 average ratio. It was expected from the non-Newtonian behaviour of seminal plasma found in [23] that individual velocities in isotonic buffer and in seminal plasma would differ more significantly. Surprisingly, the observed difference is quite tenuous. In the following, we then consider that the observed collective motion is normalized by an individual velocity 
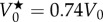
 in order to take into account the effect of the seminal plasma rheology on individual motion.

**Table 1.** Comparison between curvilinear individual spermatozoa velocity *V*_0_ obtained with a CASA, averaged over more than a hundred individual cells diluted in isotonic buffer (IB) and seminal plasma (SP).

sample no.	*V*_0_ (µm s^−1^) (IB)	*V*_0_ (µm s^−1^) (SP)	*V*_0_ (IB)/*V*_0_ (SP)
1	208.3 ± 96	127.9 ± 68	0.61
2	163.5 ± 76	145.6 ± 77	0.89
3	219.8 ± 92	170.9 ± 73	0.78
4	134.1 ± 94	95.2 ± 70	0.71
5	286.6 ± 91	198.9 ± 94	0.69

**Figure 4.**
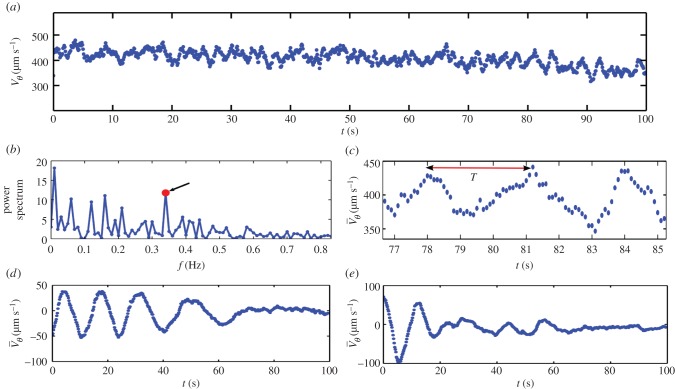
(*a*) Averaged spinning velocity 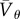 of the VS versus time for a highly active pure sample (MM score of 5 (maximal score)) at high concentration (2.96 billions ml^−1^). A slow linear decay and some temporal fluctuations can be observed. (*b*) Power spectrum of the spinning velocity fluctuation observed in (*a*). The secondary peak (red points) whose frequency is close to 0.34 Hz corresponds to the period *T* ∼ 3 s which is clearly visible on a zoom of (*a*), shown in (*c*). (*d,e*) Averaged spinning velocity 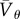 versus time for a ×4—resp. ×6—diluted sample (in isotonic buffer); in both cases, a damped oscillating spinning pattern is observed, the typical decay of which decreases for decreasing concentrations. (Online version in colour.)

This reference velocity permits one to assess whether collective effects can perform better than individual swimming. Figure 3*b* shows that this is indeed the case for *Φ* as large as 0.4. As a biological material, semen has highly variable characteristics, such as cell motility (quantified with CASA), volume fraction of dead cells, seminal plasma rheology [23] and biochemical composition. This variability induces large experimental dispersion as observed in figure 3*a*,*b*. At *Φ* larger than 0.5, each cell is in close contact with its neighbours, because the suspension almost reaches the close-packing limit. Furthermore, the VS is reached after a transient time that depends on the semen initial concentration. For pure ovine semen whose concentration ranges between 2 and 5 billions of spermatozoa per millilitre (similar ranges are found in bovine), it is almost instantaneous. Diluting the semen by a factor of 10 leads to a transient time close to few tens of seconds. When reached, the rotation can be sustained during a few minutes (cf. figure 2*c* and figure 4 or electronic supplementary material, movie S1) and slowly decays in time, almost linearly (cf. figure 2*a*). This decrease of the activity is not owing to cell death. Indeed, if we sample a small fraction of the immobile semen after 10 min, resuspend it into a fresh iso-osmotic buffer and observe it with a CASA, individual cell motion is recovered, with a similar fraction of mobile/immobile cells as in fresh semen. Furthermore, we have indirect evidence, from measurement obtained within closed chambers (unpublished data, 2015) that the decay of collective motion is related to a pH decrease, resulting from active biochemical reactions. pH is a known important factor reducing ion pump efficiency. Moreover, pH has known direct impact on individual flagellum beating [54]. So, we suggest that this mechanism is at the origin of the slow decrease of the observed collective motion. Although generic, the transition towards a VS observed in figure 3 appears variable between semen samples. This is especially true for the set of black points of figure 3*a*,*b* where much higher spinning velocities are observed and where the critical volume fraction for which rotation starts is much lower than for the other samples. We first discuss generic reasons explaining the heterogeneity of collective behaviour among semen samples and then provide more specific information about the dataset corresponding to the black points of figure 3*a*,*b*.

First, semen suspensions are far more heterogeneous than many other types of biological suspensions, such as bacterial suspensions grown under controlled incubation conditions and using selected strains. In the case of semen, the suspension constituents as well as the fluid suspension are uncontrolled. This is incidentally one motivation of this study to assess semen quality from collective motion. The cell composition (ratio of mobile/immobile cells, cell concentration, motility heterogeneity within the mobile cell population), the plasma biochemical composition as well as its rheology are variable from one semen sample to another, even if the sample comes from the same subject. Plasma rheology has been previously studied [23] showing viscosity variations as large as 50%. Two main parameters might be responsible for the observed variability: the individual cell velocity and the percentage of immobile cells. Concerning individual cell velocity variability we report in table 1 various measurements obtained for five different males, where the intrasample variability is also given. One can observe that intrasample variability has an average value of 54%, whereas inter-individual variability has an average of 58%. So, intrasample variability is comparable with inter-individual variability. The immobile cell proportion could be estimated by CASA measurements. For the samples analysed in table 1, the ratio of non-mobile/mobile sperm can vary between 20% and 40%, adding again another important contribution to the variability of collective behaviour. Finally, one should also consider that more complex behaviour may influence collective behaviour, such as the ability of sperm cells to synchronize their flagellum beating with their neighbours, but such feature is very difficult to quantify experimentally. One could wonder if more reproducible results could have been obtained by harvesting the cells from many samples and resuspending them in a fluid of known rheology mimicking semen. However, this is a highly challenging experiment to perform. Indeed, collective motility lasts no longer than half an hour that leaves a very short time window. Additionally, centrifuging has a known negative and non-controllable impact on individual motility. Finally, concentrating cells generates many cellular degradations and a large percentage of cell deaths.

Now, considering more specifically the dataset corresponding to the black points of figure 3*a*,*b*, we know from previous analyses of the seminal plasma composition as well as from the massal motility measurements of the considered ram breed (‘Lacaune’) that their plasma is highly concentrated in zinc-alpha-2-glycoprotein (ZAG) which leads to higher individual as well as collective motilities. We also know that the semen of that precise individual is highly concentrated in ZAG. This could explain why this specific dataset shows a much larger collective motility than the other ones (of the ‘Manech’ breed) presented in figure 3*a*,*b*.

We now show that these experimental observations are consistent with an interpretation in which a major role is played by polar alignment between the individual sperm cells. To support this claim, we compare the experimental results with simulations of the SOH model in a two-dimensional annular domain. As shown in §2.2, this macroscopic model is obtained from the rigorous coarse-graining of the Vicsek dynamics that describes self-propelled particles subject to polar alignment with their neighbours. Non-trivial VS steady states of (2.3)–(2.5) have been analytically obtained in (2.14). Prediction of the model is superposed with experiments results in figure 3*c*. Furthermore, from solving the set of equations (2.3)–(2.5) associated with the continuum model nearby the steady state, more information can be obtained about the active fluid behaviour. For example, figure 3*d* gives the prediction for the spinning velocity variations when changing the width of the annulus. The modelling also permits one to confirm that the VS predicted by the SOH model is stable, consistent with the experimental observations. Observation of VS has been indeed previously reported in the literature. Two references [11,12] have reported vortex-like behaviour in confined active suspensions. In [11], the appearance of vortex and counter vortex motion in droplets of *Bacillus subtilis* is reported. The droplet size is much smaller (between one and two orders of magnitude) than the annulus size of our experiments. Our work demonstrates that VS can be sustained even in large systems despite the weaker confinement, but also that the motion can set into a spontaneous systematic rotation. In [12,13], VSs are observed with colloid rollers of a few micrometre size. Here, the system is two-dimensional as the colloid beads are constrained to roll on a surface under the action of an electric force. As volume fraction increases, the system passes from a disordered state to a VS through an intermediate travelling band state. This phase has not been observed with semen. These differences may originate from the two-dimensional nature of the bead experiments, or from the long electric interaction range between the beads.

### 3.2. Dynamical behaviour

Nevertheless, in the case of very concentrated and very active semen for which MM scoring and individual velocity are the highest, we also observed more complex dynamical behaviour. Analysing quantitatively the rotation rate associated with the VS shown in figure 4*a*, we found higher-frequency spinning velocity modes superposed onto the VS mean velocity. Such dynamical behaviour is further studied from computing the Fourier decomposition (figure 4*b*) of the mean velocity temporal variations. A secondary peak in the power spectrum, associated with a typical oscillation of period *T* = 3 s (figure 4*c*) is superposed to the much larger spinning period of the VS along the annulus, *T*_0_ = 15.4 s. Similar secondary spinning modes have also been observed in diluted suspensions (see electronic supplementary material, movie S2), when the VS speed is much slower. In those cases, this secondary spinning oscillation is transient and decays after a few periods (cf. figure 4*d*,*e*). These observations are consistent with a transition toward a new oscillating state emerging from a secondary (Hopf) bifurcation from the VS, as for example occurring in Couette flows [55]. The nonlinearities of the flow can indeed trigger higher-order azimuthal oscillation modes to become stable, so that new oscillating states could branch from the first mode at high volume fractions. This qualitative interpretation is attractive to explain the observations (cf. figure 4*a*,*d*,*e*).

In figure 4*d*,*e*, a transient decay is observed for a diluted semen sample (four and six times dilution, respectively). Several possible interpretations of this transient decay can be proposed. The first one is that the corresponding volume fractions are below the threshold for the secondary bifurcation, whereas the self-sustained oscillations (figure 4*a*) would correspond to a volume fraction above this threshold. Because following this interpretation the system stands below the transition, the initiation of this damped rotation could be owing to the initial conditions. Indeed, the set-up is initiated by manually depositing the top cover onto the sessile semen droplet laid onto the annular channel. The lateral motion of the top cover could provide the initial kick for the motion to be initiated. Another interpretation could be that the corresponding volume fractions are slightly above this secondary threshold, but only slightly in such a way that the rotation speeds are rather small (they are 10 times below the speed of the pure semen in figure 4*a*). In this case, the motion might be sensitive to the degradation of the environment such as pH increase owing to the activity of the cells. A slow degradation would eventually lead the sample to cross the threshold and return to a steady rotation state. This oscillating state has been observed both for samples diluted in isotonic buffer and in seminal plasma (not shown). We did not find any reported observation of spontaneous reversal of collective rotation for other types of active suspensions. Our observations bear intriguing similarities with experiments on locust behaviour [18] and could originate from strong fluctuations in sperm orientation.

### 3.3. Rotation speed versus mass motility score

**Figure 5.**
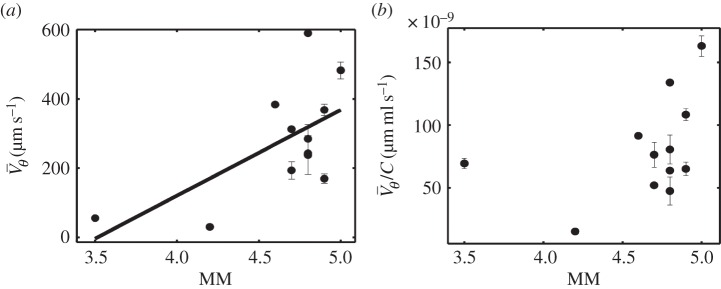
(*a*) Each point represents the averaged spinning velocity 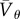 of a pure fresh semen sample versus the MM score for different samples. A linear regression has been performed on the data, the result of which is plotted with a continuous line. (*b*) Averaged spinning velocity divided by concentration versus MM score.

The observed rotation in ring-shaped arenas can be used to assess the motility and the concentration of the active fluid, and in the case of semen, the rotation speed provides an interesting criterion to assess its quality. Here, we question the correlation of the rotation speed in the annulus with the MM score currently used in insemination centres. Previous statistical studies [56,57] have indeed suggested the existence of a positive correlation between fertility and the MM scoring of semen. For each sample, we perform in parallel MM scoring and rotation speed measurement inside the annulus. As shown in figure 5*a*, there is a good correlation between the MM scores and the spinning velocity. The continuous line of figure 5*a* provides the result of a linear model. A Student's *t*-test built with the null hypothesis of a zero slope provides a significant result whose *p*-value is *p* = 0.03, which strengthens the hypothesis of the existence of a linear correlation between the rotation velocity and the MM index.

Finally, from figure 5*b*, we also note that the spinning velocity normalized by the concentration increases with MM score for MM scores above 4. For the sake of classification of ejaculates according to their MM, this method can provide an interesting possibility [58].

## 4. Conclusion

In this contribution, we show that concentrated active suspension of pushers (spermatozoa), when confined into an annular microfluidic device, display non-trivial spontaneous motion. A phase transition associated with a rotational motion arises at a critical volume fraction of active cells, and has been experimentally studied. At higher volume fraction, more complex dynamical states associated with secondary oscillating rotation have been observed and described. A macroscopic continuum model based upon SOH theory provides predictions consistent with the observed stationary motion. We hope that the proposed set-up could be useful, on the one hand, from the fundamental viewpoint to characterize active suspensions, and on the other hand, to provide interesting simple quantification of semen collective motion. Further studies are needed to identify the detailed mechanism by which the cells align, resulting from the possibly combined contributions of steric interactions, soft lubrication between cell bodies and flagellum beating interactions.

## Data accessibility

Data supporting this work are available on Dryad: http://dx.doi.org/10.5061/dryad.7309b [59].

## References

[1] Koch DL, Subramanian G 2011 Collective hydrodynamics of swimming microorganisms: living fluids. Annu. Rev. Fluid Mech. 43, 637–659. (10.1146/annurev-fluid-121108-145434)

[2] Ramaswamy S 2010 The mechanics and statistics of active matter. Annu. Rev. Condens. Matter Phys. 1, 323–345. (10.1146/annurev-conmatphys-070909-104101)

[3] Elgeti J, Winkler RG, Gompper G 2015 Physics of microswimmers—single particle motion and collective behavior: a review. Rep. Prog. Phys. 78, 056601 (10.1088/0034-4885/78/5/056601) 25919479

[4] Saintillan D 2010 Extensional rheology of active suspensions. Phys. Rev. E 81, 056307 (10.1103/PhysRevE.81.056307) 20866322

[5] Rafa S, Jibuti L, Peyla P 2010 Effective viscosity of microswimmer suspensions. Phys. Rev. Lett. 104, 098102 (10.1103/PhysRevLett.104.098102) 20367014

[6] Lauga E, Powers TR 2009 The hydrodynamics of swimming microorganisms. Rep. Prog. Phys. 72, 096601 (10.1088/0034-4885/72/9/096601)

[7] Gaffney E, Gadêlha H, Smith D, Blake J, Kirkman-Brown J 2011 Mammalian sperm motility: observation and theory. Annu. Rev. Fluid Mech. 43, 501–528. (10.1146/annurev-fluid-121108-145442)

[8] Denissenko P, Kantsler V, Smith DJ, Kirkman-Brown J 2012 Human spermatozoa migration in microchannels reveals boundary-following navigation. Proc. Natl Acad. Sci. USA 109, 8007–8010. (10.1073/pnas.1202934109) 22566658 PMC3361448

[9] Tung C-K, Ardon F, Fiore AG, Suarez SS, Wu M 2014 Cooperative roles of biological flow and surface topography in guiding sperm migration revealed by a microfluidic model. Lab Chip 14, 1348–1356. (10.1039/C3LC51297E) 24535032 PMC4497544

[10] Kantsler V, Dunkel J, Blayney M, Goldstein RE 2014 Rheotaxis facilitates upstream navigation of mammalian sperm cells. eLife 3, e02403 (10.7554/elife.02403) 24867640 PMC4031982

[11] Wioland H, Woodhouse FG, Dunkel J, Kessler JO, Goldstein RE 2013 Confinement stabilizes a bacterial suspension into a spiral vortex. Phys. Rev. Lett. 110, 268102 (10.1103/PhysRevLett.110.268102) 23848925

[12] Bricard A, Caussin JB, Desreumaux N, Dauchot O, Bartolo D 2013 Emergence of macroscopic directed motion in populations of motile colloids. Nature 503, 95–98. (10.1038/nature12673) 24201282

[13] Bricard A et al. 2015 Emergent vortices in populations of colloidal rollers. Nat. Commun. 6, 7470 (10.1038/ncomms8470) 26088835 PMC4557359

[14] Dombrowski C, Cisneros L, Chatkaew S, Goldstein RE, Kessler JO 2004 Self-concentration and large-scale coherence in bacterial dynamics. Phys. Rev. Lett. 93, 098103 (10.1103/PhysRevLett.93.098103) 15447144

[15] Riedel IH, Kruse K, Howard J 2005 A self-organized vortex array of hydrodynamically entrained sperm cells. Science 309, 300–303. (10.1126/science.1110329) 16002619

[16] Smyth P, Gordon I 1967 Seasonal and breed variations in the semen characteristics of rams in Ireland. Irish Vet. 21, 222.

[17] Herman HA, Madden FW 1947 The artificial insemination of dairy cattle, a handbook and laboratory manual. Columbia: MO, Lucas Brothers.

[18] Buhl J, Sumpter DJT, Couzin ID, Hale JJ, Despoland E, Miller ER, Simpson SJ 2006 From disorder to order in marching locusts. Science 312, 1402–1406. (10.1126/science.1125142) 16741126

[19] Kelley DH, Ouellette NT 2013 Emergent dynamics of laboratory insect swarms. Sci. Rep. 3, 1073 (10.1038/srep01073) 23323215 PMC3545223

[20] Zhang H, Beér A, Florin EL, Swinney HL 2010 Collective motion and density fluctuations in bacterial colonies. Proc. Natl Acad. Sci. USA 107, 13 626–13 630. (10.1073/pnas.1001651107) PMC2922251 20643957

[21] Zhang J, Tan KL, Hong GD, Yang LJ, Gong HQ 2001 Polymerization optimization of SU-8 photoresist and its applications in microfluidic systems and MEMS. J. Micromech. Microeng. 11, 20–26 (10.1088/0960-1317/11/1/304)

[22] Lin C-H, Lee G-B, Chang B-W, Chang G-L 2002 A new fabrication process for ultra-thick microfluidic microstructures utilizing SU-8 photoresist. J. Micromech. Microeng. 12, 590–597. (10.1088/0960-1317/12/5/312)

[23] Creppy A, Praud O, Druart X, Kohnke PL, Plouraboué F 2015 Turbulence of swarming sperm. Phys. Rev. E 92, 032722 (10.1103/PhysRevE.92.032722) 26465513

[24] Nobach H, Honkanen M 2005 Two-dimensional Gaussian regression for sub-pixel displacement estimation in particle image velocimetry or particle position estimation in particle tracking velocimetry. Exp. Fluids 38, 511–515. (10.1007/s00348-005-0942-3)

[25] Takehara K, Etoh T 1999 A study on particle identification in PTV particle mask correlation method. J. Visual. 1, 313–323. (10.1007/BF03181412)

[26] Saintillan D, Shelley MJ 2008 Instabilities, pattern formation, and mixing in active suspensions. Phys. Fluids 20, 123304 (10.1063/1.3041776) 18518342

[27] Abkenar M, Marx K, Auth T, Gompper G 2013 Collective behavior of penetrable self-propelled rods in two dimensions. Phys. Rev. E 88, 062314 (10.1103/PhysRevE.88.062314) 24483451

[28] Peruani F, Deutsch A, Bär M 2006 Nonequilibrium clustering of self-propelled rods. Phys. Rev. E 74, 030904(R). (10.1103/PhysRevE.74.030904) 17025586

[29] Wensink HH, Dunkel J, Heidenreich S, Drescher K, Goldstein RE, Lowen H, Yeomans JM 2012 Meso-scale turbulence in living fluids. Proc. Natl Acad. Sci. USA 109, 14 308–14 313. (10.1073/pnas.1202032109) PMC3437854 22908244

[30] Peruani F, Deutsch A, Bär M 2008 A mean-field theory for self-propelled particles interacting by velocity alignment mechanisms. Eur. Phys. J. Special Top. 157, 111–122. (10.1140/epjst/e2008-00634-x)

[31] Ginelli F, Peruani F, Bär M, Chaté H 2010 Large-scale collective properties of self-propelled rods. Phys. Rev. Lett. 104, 184502 (10.1103/PhysRevLett.104.184502) 20482178

[32] Peshkov A, Aranson IS, Bertin E, Chaté H, Ginelli F 2012 Nonlinear field equations for aligning self-propelled rods. Phys. Rev. Lett. 109, 268701 (10.1103/PhysRevLett.109.268701) 23368625

[33] Vicsek T, Czirok A, Ben-Jacob E, Cohen I, Shochet O 1995 Novel type of phase transition in a system of self-driven particles. Phys. Rev. Lett. 75, 1226–1229. (10.1103/PhysRevLett.75.1226) 10060237

[34] Vicsek T, Zafeiris A 2012 Collective motion. Phys. Rep. 517, 71–140. (10.1016/j.physrep.2012.03.004)

[35] Degond P, Motsch S 2008 Continuum limit of self-driven particles with orientation interaction. Math. Models Methods Appl. Sci. 18, 1193–1215. (10.1142/S0218202508003005)

[36] Degond P, Frouvelle A, Liu JG 2015 Phase transitions, hysteresis, and hyperbolicity for self-organized alignment dynamics. Arch. Ration. Mech. Anal. 216, 63–115. (10.1007/s00205-014-0800-7)

[37] Degond P, Dimarco G, Mac TBN, Wang N 2015 Macroscopic models of collective motion with repulsion. Commun. Math. Sci. 13, 1615–1638. (10.4310/CMS.2015.v13.n6.a12)

[38] Degond P, Yu H 2015 Self-organized hydrodynamics in an annular domain: modal analysis and nonlinear effects. Math. Models Methods Appl. Sci. 25, 495–519. (10.1142/S0218202515400047)

[39] Toner J, Tu Y 1995 Long-range order in a two-dimensional dynamical XY model: how birds fly together. Phys. Rev. Lett. 75, 4326–4329. (10.1103/PhysRevLett.75.4326) 10059876

[40] Bolley F, Cañizo JA, Carrillo JA 2011 Mean-field limit for the stochastic Vicsek model. Appl. Math. Lett. 25, 339–343. (10.1016/j.aml.2011.09.011)

[41] Tu Y, Toner J, Ulm M 1998 Sound waves and the absence of Galilean invariance in flocks. Phys. Rev. Lett. 80, 4819–4822. (10.1103/PhysRevLett.80.4819)

[42] Motsch S, Navoret L 2011 Numerical simulations of a nonconservative hyperbolic system with geometric constraints describing swarming behavior. Multiscale Model. Simul. 9, 1253–1275. (10.1137/100794067)

[43] Toner J, Tu Y 1998 Flocks, herds, and schools: a quantitative theory of flocking. Phys. Rev. E 58, 4828–4858. (10.1103/PhysRevE.58.4828)

[44] Toner J, Tu Y, Ramaswamy S 2005 Hydrodynamics and phases of flocks. Ann. Phys. 318, 170–244. (10.1016/j.aop.2005.04.011)

[45] Degond P, Yang T 2010 Diffusion in a continuum model of self-propelled particles with alignment interaction. Math. Models Methods Appl. Sci. 20, 1459–1490. (10.1142/S0218202510004659)

[46] Degond P, Liu J-G, Motsch S, Panferov V 2013 Hydrodynamic models of self-organized dynamics: derivation and existence theory. Methods Appl. Anal. 20, 89–114. (10.4310/maa.2013.v20.n2.a1)

[47] Marchetti MC, Joanny JF, Ramaswamy S, Liverpool TB, Prost J, Rao M, Simha RA 2013 Hydrodynamics of soft active matter. Rev. Mod. Phys. 85, 1143–1189. (10.1103/RevModPhys.85.1143)

[48] Bertin E, Droz M, Grégoire G 2009 Hydrodynamic equations for self-propelled particles: microscopic derivation and stability analysis. J. Phys. A Math. Theor. 42, 445001 (10.1088/1751-8113/42/44/445001)

[49] Carlen EA, Carvalho MC, Degond P, Wennberg B 2015 A Boltzmann model for rod alignment and schooling fish. Nonlinearity 28, 1783–1803. (10.1088/0951-7715/28/6/1783)

[50] Degond P, Frouvelle A, Raoul G 2014 Local stability of perfect alignment for a spatially homogeneous kinetic model. J. Stat. Phys. 157, 84–112. (10.1007/s10955-014-1062-3)

[51] Jiang N, Xiong L, Zhang T-F 2015 Hydrodynamic limits of the kinetic self-organized models. Beijing, China: Tsinghua University.

[52] Degond P, Henkes S, Yu H 2016 Self-organized hydrodynamics with nonconstant velocity. (http://arxiv.org/abs/1602.06195)

[53] Frouvelle A 2012 A continuum model for alignment of self-propelled particles with anisotropy and density-dependent parameters. Math. Models Methods Appl. Sci. 22, 1250011 (10.1142/S021820251250011X)

[54] Gatti J-L, Chevrier C, Paquignon M, Dacheux J-L 1993 External ionic conditions, internal pH and motility of ram and boar spermatozoa. J. Reproduct. Fertil. 98, 439–449. (10.1530/jrf.0.0980439) 8410809

[55] Mamun C, Tuckerman L 1995 Asymmetry and Hopf bifurcation in spherical Couette flow. Phys. Fluids 7, 80–91. (10.1063/1.868730)

[56] David I, Bodin L, Lagriffoul G, Leymarie C, Manfredi E, Robert-Granié C 2007 Genetic analysis of male and female fertility after artificial insemination in sheep: comparison of single-trait and joint models. J. Dairy Sci. 90, 3917–3923. (10.3168/jds.2006-764) 17639003

[57] David I, Druart X, Lagriffoul G, Manfredi E, Robert-Granié C, Bodin L 2007 Genetic and environmental effects on semen traits in Lacaune and Manech tete rousse AI rams. Genet. Sel. Evol. 39, 405–419. (10.1186/1297-9686-39-4-405) 17612480 PMC2682819

[58] Degond P, Plouraboué F, Praud O, Creppy A 2014 *Device for the quality assessment of an active fluid sample*. Patent no. PCT/EP2014/075503.

[59] Creppy A, Plouraboué F, Praud O, Druart X, Cazin S, Yu H, Degond P 2016 Data from: Symmetry-breaking phase transitions in highly concentrated semen. Dryad Digital Repository. (10.5061/dryad.7309b) PMC5095218 27733694

